# Memory Deficit Recovery after Chronic Vanadium Exposure in Mice

**DOI:** 10.1155/2016/4860582

**Published:** 2016-01-19

**Authors:** Oluwabusayo Folarin, Funmilayo Olopade, Silas Onwuka, James Olopade

**Affiliations:** ^1^Neuroscience Unit, Department of Veterinary Anatomy, University of Ibadan, Ibadan 20001, Nigeria; ^2^Department of Anatomy, Ladoke Akintola University, Ogbomosho 21001, Nigeria; ^3^Department of Anatomy, University of Ibadan, Ibadan 20001, Nigeria

## Abstract

Vanadium is a transitional metal with an ability to generate reactive oxygen species in the biological system. This work was designed to assess memory deficits in mice chronically exposed to vanadium. A total of 132 male BALB/c mice (4 weeks old) were used for the experiment and were divided into three major groups of vanadium treated, matched controls, and animals exposed to vanadium for three months and thereafter vanadium was withdrawn. Animals were tested using Morris water maze and forelimb grip test at 3, 6, 9, and 12 months of age. The results showed that animals across the groups showed no difference in learning but had significant loss in memory abilities after 3 months of vanadium exposure and this trend continued in all vanadium-exposed groups relative to the controls. Animals exposed to vanadium for three months recovered significantly only 9 months after vanadium withdrawal. There was no significant difference in latency to fall in the forelimb grip test between vanadium-exposed groups and the controls in all age groups. In conclusion, we have shown that chronic administration of vanadium in mice leads to memory deficit which is reversible but only after a long period of vanadium withdrawal.

## 1. Introduction

There has been an increased awareness of accumulating levels of toxic metals in the atmosphere and their relationship with health [1–4]. More pertinent in this regard is the increasing wave of neurodegenerative diseases and the roles that metal exposure may be playing in the pathogenesis of these diseases [5]. Vanadium is a transitional metal of atomic number 23. The metal has outer orbitals that contain eleven electrons in shell 3 and two electrons in shell 4. This arrangement allows for numerous electronic exchange reactions and consequently the formation of a wide range of organic and inorganic complexes that contains vanadium in different oxidation states [6]. Vanadium and its agents are important in several industrial processes. Vanadium is used to improve the hardness, malleability, and fatigue resistance of steel [7]; and vanadium alloys are highly valued in the production of aerospace products [8]. In addition, vanadium agents are utilised in the production of glass, pigments, varnishes, reducing agents, and inks [8] and in fertilizers production [9] amongst others. Vanadium is widely distributed in the environment [10] with exposure to the metal occurring through vanadium mining sites and as contaminants during the mining of some heavy metals [8]. In addition, vanadium is released into the atmosphere through forest fires, volcanic emissions, and burning of fossil fuels in vanadium-contaminated crude as seen in Venezuela, the Arabian Gulf, the Gulf of Mexico, and the Nigerian Niger Delta [1, 6, 11]. Vanadium has been reported as the most abundant trace metal in petroleum samples [12] and accumulates in the soil, groundwater, and vegetation, being consumed by animals and humans [13]. The study of the neurotoxic profile of vanadium has been on the rise in recent years [14–17]. Behavioral changes associated with vanadium exposure include lethargy, tremor, anger hostility, depression-dejection, and various locomotor deficits [10, 14, 15]. In fact, Naylor [18] reported raised levels of vanadium in different body samples of manic and depressed patients while Avila-Costa et al. [19] reported time dependent loss of dendritic spines, necrotic like cell death, and profound alterations in the hippocampal CA1 neuropile. Few studies have however reported progressive memory loss induced by vanadium exposure. Most animal experiments on behavioral deficits involving vanadium exposure have been based on acute exposure while in reality many patients occupationally [6] and environmentally [1] exposed to vanadium are so exposed for decades or even a life time. This work is designed to assess memory deficits in rats chronically exposed to vanadium.

## 2. Materials and Methods

### 2.1. Animal Design

A total of 132 male BALB/c mice (4 weeks old) were used for the experiment which covered a period of 12 months. The animals were assigned to one of the following animal groups: vanadium- (V-) treated, withdrawal, and control groups. Animal experiments were done in accordance with University of Ibadan Ethical Research Committee guidelines for use of research animals. V-treated group consisted of four subgroups of twelve separate animals each. The subgroups are designated as VAN3, VAN6, VAN9, and VAN12. The mice (from 4 weeks old) were injected with 3 mg/kg b.w./day of vanadium (sodium metavanadate), i.p. thrice a week for 3, 6, 9, and 12 months. After the treatment, the animals in each group (*n* = 12) were subjected to behavioral analysis which included test for cognitive and memory function by using Morris water maze and forelimb grip strength tests; these were carried out on every animal in each group. Withdrawal group consisted of a total of three groups of 12 animals each. The subgroups are designated as W3, W6, and W9. The mice (from 4 weeks old) were injected with 3 mg/kg b.w./day of vanadium (sodium metavanadate), i.p. thrice a week only for the first three months and then vanadium administration was stopped. After the treatment, the animals in each group were subjected to behavioral analysis which included tests for cognitive and memory function as mentioned earlier 3(W3), 6(W6), and 9(W9) months after the vanadium treatment had been withdrawn. Control group consisted of four subgroups of twelve different animals each. The subgroups are designated as C3, C6, C9, and C12. The mice (from 4 weeks old) were injected with sterile water, i.p. thrice a week for 3, 6, 9, and 12 months which was volume matched with the V-treated group. After the treatment, the animals were subjected to behavioral analysis which included test for cognitive and memory function as mentioned earlier in V-treated group.

### 2.2. Behavioral Tests

#### 2.2.1. Forelimb Grip Strength Test

This test involves the forepaws of the mice being placed on a horizontally suspended wire (measuring 2 mm in diameter and 1 m in length), placed one meter above a soft bedding-filled landing area. The latency to fall (i.e., length of time each mice was able to stay suspended before falling off the wire) was recorded with a stopwatch. A maximum time of 2 minutes was given to each mouse after which it was removed and each mouse had two trials. This test reflects muscular strength and balance in the animals [20, 21].

#### 2.2.2. Modified Morris Water Maze Test

The modified Morris water maze is a circular pool of water (110 cm in diameter, 30 cm in height) with a hidden circular escape platform (12 cm in diameter) which the mouse must learn its location using contextual and visual cues. This tests hippocampal-dependent spatial learning and memory in rodents [22, 23]. The task is based on the principle that rodents are highly motivated to escape from a water environment by the fastest, most direct route. The pool was marked North, South, East, and West and the hidden platform was placed in a particular spot. Each mouse was dropped into the pool and expected to find the platform, and the length of time it takes to find the platform was recorded. If it did not find the platform after 60 seconds, the mouse was guided to the platform and allowed to stay there for 15 seconds. Each mouse went through three trials per day for two consecutive days. This test is a measure of the learning ability of the mouse. On the third day, a single probe trial was given to test the mouse's spatial memory in the water maze while the platform was removed. Its memory of the initial location of the escape platform was tested by measuring the time it spent in the target quadrant and the number of times it crossed the island zone where the platform was initially located. This latter record is a test of its memory ability. The test was recorded manually by two observers using a stopwatch. The Morris water maze was introduced as an instrument with particular sensitivity to the effects of hippocampal lesions in rodents [23, 24].

## 3. Results

**Figure 1:**
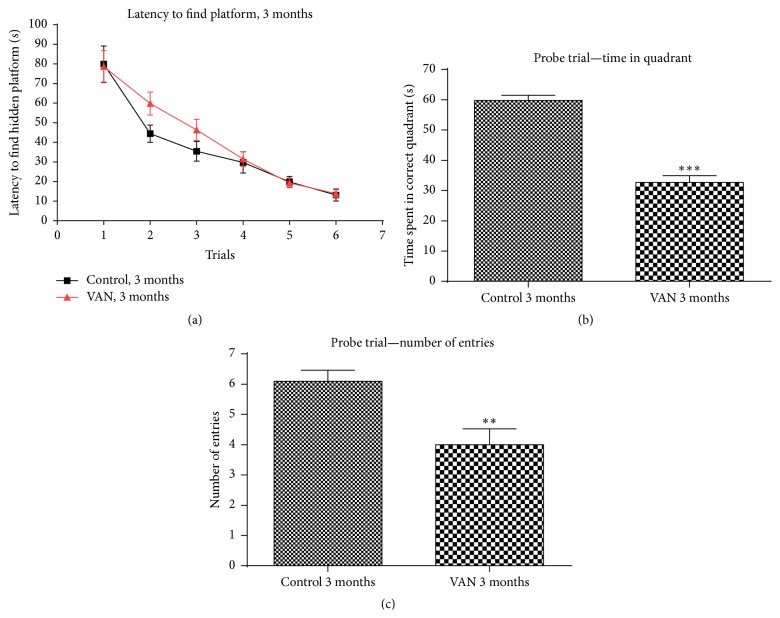
Effect of intermittent vanadium (VAN) treatment for three months on learning and memory in mice: 12 male mice were exposed thrice a week for three months to VAN (3 mg/kg b.w.) with equal number and age matched controls. The ability to learn the location of the hidden platform was unperturbed because they gradually spent shorter times to locate the platform with successive training trials. There was no statistically significant difference between the groups in their learning ability (a) however; the length of time in target quadrant (^*∗∗∗*^ *P* < 0.001) and annulus crossings of the VAN group (^*∗∗*^ *P* < 0.01) were significantly decreased when compared to the controls ((b) and (c)).

**Figure 2 fig2:**
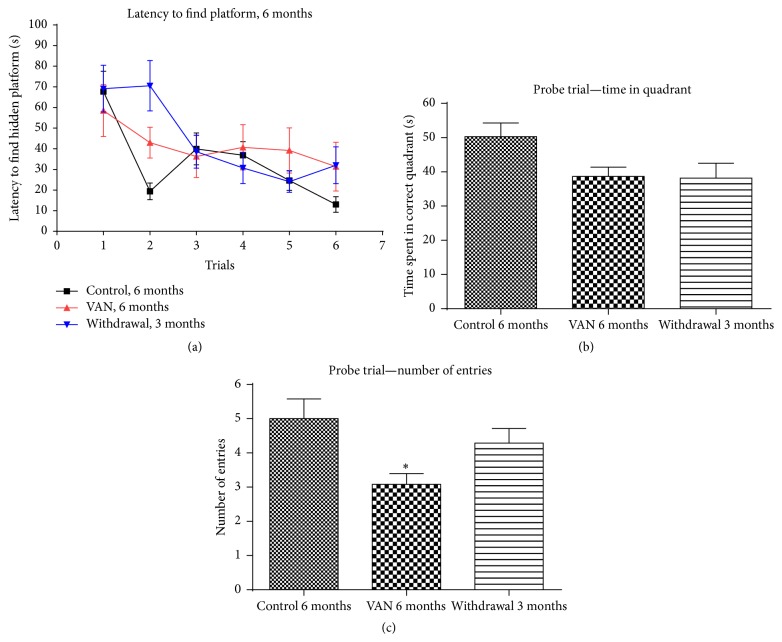
Effect of intermittent vanadium (VAN) treatment for six months on learning and memory in mice: 12 male mice were exposed thrice a week for six months to VAN (3 mg/kg b.w.) with equal number and age matched controls. The withdrawal group were exposed to VAN for three months but replaced with water thereafter. All the groups of the 6-month-old mice (C6, VAN6, and W3) were able to learn the location of the hidden platform and there was no statistically significant difference among the groups in their ability to learn (a) however; the memory retention of the V6 group was significantly decreased when compared to the control group (^*∗*^ *P* < 0.05) ((b) and (c)).

**Figure 3 fig3:**
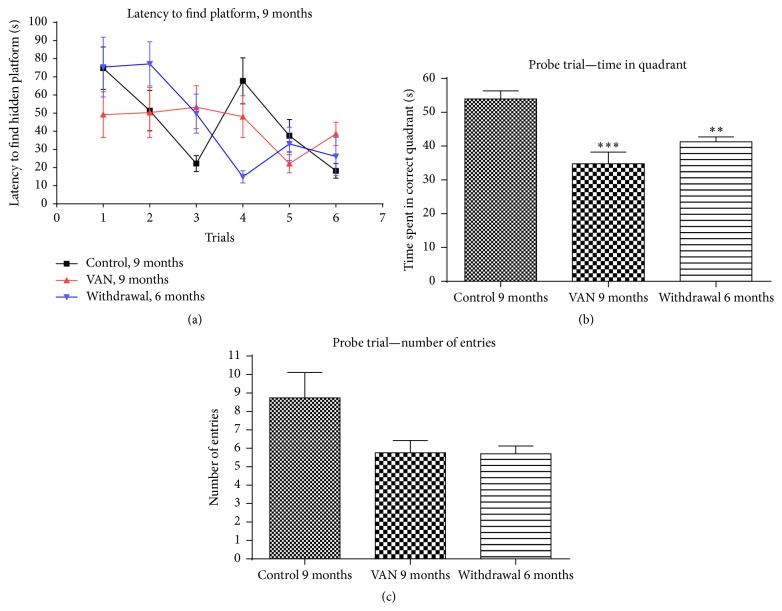
Effect of intermittent vanadium (VAN) treatment for nine months on learning and memory in mice: 12 male mice were exposed thrice a week for nine months to VAN (3 mg/kg b.w.) with equal number and age matched controls. The withdrawal group were exposed to VAN for three months but replaced with water thereafter. All the groups of the 9-month-old rats (C9, VAN9, and W6) were able to learn the location of the hidden platform with no statistically significant difference among them (a). However, there was a statistically significant decrease in memory retention in vanadium (^*∗∗∗*^ *P* < 0.001) and withdrawal (^*∗∗*^ *P* < 0.01) groups relative to control ((b) and (c)). The W6 group showed some recovery ((b) and (c)) in comparison to the VAN9 group but was still significantly less than the controls.

**Figure 4 fig4:**
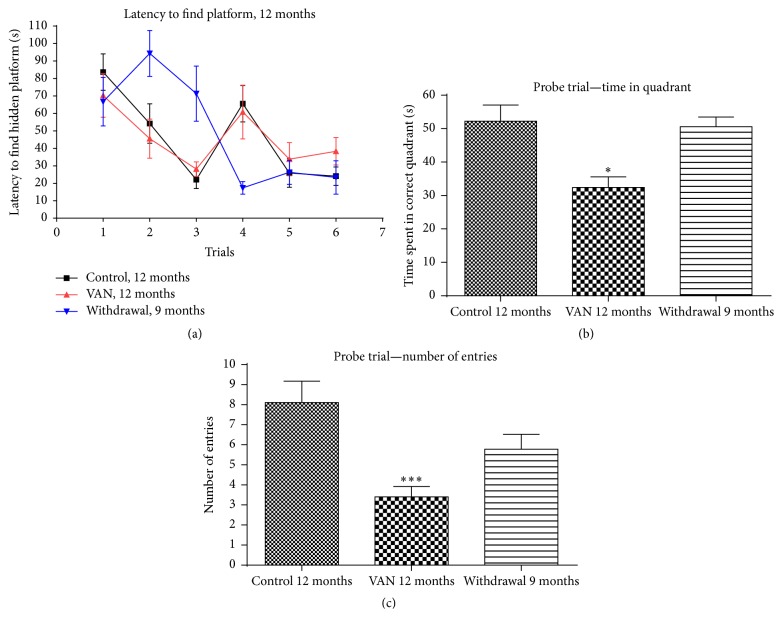
Effect of intermittent vanadium (VAN) treatment for twelve months on learning and memory in mice: 12 male mice were exposed thrice a week for twelve months to VAN (3 mg/kg b.w.) with equal number and age matched controls. The withdrawal group were exposed to VAN for three months but replaced with water thereafter. All the groups of the 12-month-old rats (C12, V12, and W9) were able to learn the location of the hidden platform with no statistically significant difference among them. However, there was statistically significant decrease in memory retention in V12 (^*∗*^ *P* < 0.05 and ^*∗∗∗*^ *P* < 0.001) relative to controls ((b) and (c)). The W9 group showed a remarkable recovery in memory retention being significantly higher (*P* < 0.01) than the V12 (b) and were comparable to the controls.

**Figure 5:**
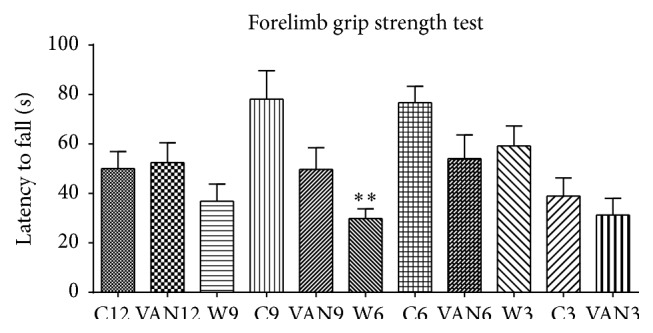
Effect of intermittent vanadium (VAN) treatment for twelve months on learning and memory in mice: 12 male mice each were exposed thrice a week for three, six, nine, and twelve months to VAN (3 mg/kg b.w.) with equal number and age matched controls. The withdrawal group were exposed to VAN for three months but replaced with water thereafter. All the groups of 3–12-month-old mice were subjected to forelimb grip test with 6–12 months reported here. There was no significant difference in latency to fall in the forelimb grip test between vanadium-exposed groups and their respective controls in all age groups. There was a significant reduction in latency to fall in vanadium withdrawal group W6 (^*∗∗*^ *P* < 0.01) relative to their controls.

The results obtained in this study are as described in Figures 1 [Fig fig2] [Fig fig3] [Fig fig4]–5. Animals across the groups show no difference in learning abilities but significant loss in memory abilities after 3 months of vanadium exposure and this trend continued in all vanadium-exposed groups relative to control (Figures 1–4). Animals exposed to vanadium for three months after which treatment was withdrawn recovered significantly only 9 months after vanadium withdrawal. There was no significant difference in latency to fall in the forelimb grip test between vanadium-exposed groups and their respective controls in all age groups. There was however a significant reduction in latency to fall in vanadium withdrawal group W6 (nine months old) relative to their controls (Figure 5).

## 4. Discussion

In this study, vanadium administration led to significant loss in memory abilities as early as 3 months after exposure and this was consistent till 12 months. Previous studies using the Morris water maze have reported reduced memory scores after metal exposures. Haider et al. [25] showed a dose dependent impairment in memory function in cadmium-treated rats relative to controls and Lu et al. [26] demonstrated significantly longer escape latency in manganese exposed rats when compared to controls. There are reports of behavioral deficits after vanadium exposure [16, 17, 27]; however, few have reported on memory deficits using the Morris water maze. In addition, our data showed that long term vanadium exposure (3–12 months) leads to significant memory deficits which persist long after exposure has stopped. This could have implications for people who are occupationally or environmentally exposed to vanadium over a long period of time. Of note is the huge population in the Arabian Gulf and Nigerian Niger Delta who have been exposed for decades to vanadium-contaminated crude burning in relatively confined ecosystem [1]. A striking observation in this study is the significant improvement in memory scores observed in mice exposed to vanadium for 3 months after a withdrawal period of 9 months, but not earlier. This implies the reversibility of vanadium mediated effects in the brain after vanadium withdrawal, despite being only after a long withdrawal period. A reversal of tissue damaging effects (not the brain) of vanadium after withdrawal has been reported by Olopade et al. [28]. Barker et al. [29] reported that previous benzodiazepine users will likely experience benefits of improved cognitive functioning after withdrawal but will not experience full restitution of functions; similarly, deficits resulting from vanadium exposure though reversible could take a long time before full restitution is obtained. Also, observed in this study was the fact that although vanadium exposure led to memory deficits, the ability of the mice to learn was largely unaffected. All the groups of mice were capable of learning the spatial location of the escape platform, shown by the progressive reduction observed in escape latency with subsequent trials. However, in contrast to controls, the vanadium-exposed mice were impaired in their ability to remember the escape position during the probe trial. Impaired spatial retention of vanadium-exposed mice during the probe trials was observed by comparison of annulus crossings over the trained position and time spent in trained quadrant. The Morris water maze (MWM) is a test of spatial learning which is assessed across repeated trials and reference memory which is determined by preference for the platform area when the platform is absent (probe trial). There is extensive evidence of its validity as a measure of hippocampal-dependent spatial navigation and reference memory [30] and its specificity as a measure of place learning. Consolidation of memory requires a fully functional hippocampus which converts the working/short term memory into long term memory [31]. This long term potentiation seems to be the focus of neurological deficit observed in vanadium-exposed mice as they showed a significantly reduced ability to consolidate the memory of the location of the escape platform. Consolidation of memory is dependent on phosphorylation of hippocampal mitogen activated protein kinase (MAPK)/ERK and subsequent synaptic plasticity [32]. Standing on the premises that hippocampal ERK phosphorylation has been shown to be required during recent spatial learning and memory, Leon et al. [33] reported that its inhibition leads to failure of consolidation of memory, though it did not affect spatial acquisition. We propose that the deficit in memory consolidation observed in vanadium-exposed mice is due to a disruption in this process. Vanadium exposure has been found to lead to the loss of dendritic spines and necrotic-like cell death in the hippocampus [19] and this could also be the possible cause. The recovery seen in withdrawal group after 9 months could be related to adult neurogenesis or a plastic reorganization of neuronal networks compensating for possible early neuronal losses [34]. The forelimb grip test did not show any significant changes in the vanadium-exposed animals relative to controls. Though forelimb grip test has shown reduced muscular strength after vanadium exposure, the difference was not significant. It seems that long term vanadium exposure may not affect muscular activity and coordination to a large degree. A human study by Charles et al. [35] did not provide evidence that occupational exposure to pesticides, solvents, and metals adversely affected hand-grip strength in the studied population. The histological analysis of the different brain regions of these mice is ongoing in our laboratory and this will hopefully shed more light on the pathogenesis of memory loss seen in this study. In conclusion, we have shown that chronic administration of vanadium in mice leads to memory deficits which are reversible only after a long period of vanadium withdrawal.

## 5. Conclusion

This work has shown that mice exposed to vanadium over a period of time exhibited no difference in learning abilities but had significant loss in memory acumen after 3 months of exposure.

Our work also revealed that the memory deficit induced by chronic administration of vanadium in mice is reversible but only after a long period of vanadium withdrawal.
